# Preparation of Dispersed Particle Gel (DPG) through a Simple High Speed Shearing Method

**DOI:** 10.3390/molecules171214484

**Published:** 2012-12-06

**Authors:** Caili Dai, Guang Zhao, Mingwei Zhao, Qing You

**Affiliations:** 1 State Key Laboratory of Heavy Oil Processing, China University of Petroleum, Qingdao 266580, Shandong, China; E-Mail: zhaomingwei@upc.edu.cn; 2 School of Earth and Space Sciences, Peking University, Beijing 100871, China; E-Mail: youqing_dandong@yahoo.com.cn

**Keywords:** dispersed particle gel, high speed shearing, colloid mill

## Abstract

Dispersed particle gel (DPG) has been first successfully prepared using cross-linked gel systems through a simple high speed shearing method with the aid of a colloid mill at room temperature. The gel microstructure and particle size were investigated by scanning electron microscope (SEM), transmission electron microscope (TEM), and dynamic light scattering (DLS) measurements. The results clearly show that the prepared DPG particles have highly uniformly spherical structures with an average size of 2.5 μm. A possible mechanism for the formation of DPG has been put forward and discussed in details. The high speed shearing method is considered to be the simple and rapid method for the preparation of DPG.

## 1. Introduction

Particle gels with controllable size such as preformed particle gels [1], branched preformed particle gels [2], pore-scale elastic microspheres [3], and microgels [4,5] have been developed very rapidly due to their wide applications in oilfield for in-depth profile control and water shut-off treatments. The particle gels can be obtained through many different methods, including emulsion polymerization [6,7], precipitation polymerization [2], shearing cross-linking by peristaltic pump [4] or coaxial cylinder viscometer [5] and so on. However, the polymerizations involved in the synthesis of pore-scale elastic microspheres or PPG with monomers or *N,N*-dimethylaniline which can cause damage to the environment in the production process can’t meet the current environmental regulations [8]. In addition, due to the smaller displacement and lower production efficiency, the other preparation methods, such as shearing cross-linking by peristaltic pump or coaxial cylinder viscometer, can’t meet the requirements of large-scale production, which limits the development of this technology application in the oilfield.

In this work, to satisfy the industry demands and protect the environment, a simple preparation method is firstly proposed. The dispersed particle gel (DPG) can be prepared at room temperature by a colloid mill with high speed shearing using cross-linked gel systems. The cross-linked gel system formed by polyacrylamide and phenolic resin are chosen to prepare the DPG, due to its many excellent properties, such as thermal stability, good viscoelasticity, salt durability (salinity tolerance) and environmentally friendly nature, which were investigated thoroughly by our group [9,10]. More important is the fact that the colloid mill methods with high production efficiency can meet the requirements of large-scale production, which can break the bulk gel systems into particles with very small sizes by imposing high speed shearing forces upon the material in several minutes. In order to provide a more complete understanding of the DPG, it is necessary to study the gel microstructure and particle size. The mechanism for the formation of DPG is also proposed. We expect these results can help to overcome the limitations of the common methods for the preparation of DPG.

## 2. Results and Discussion

**Figure 1:**
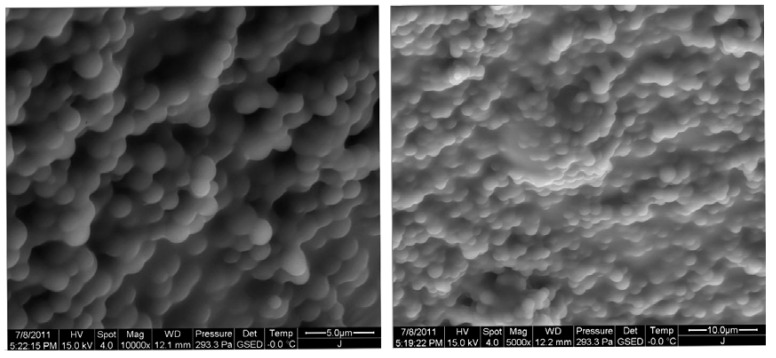
ESEM images of the bulk gel before treatment.

In order to study the microstructure and morphology of the prepared DPG particles, ESEM was conducted to investigate the microstructure of the bulk gel systems before shearing treatment [11]. Figure 1 shows the ESEM images of the microstructure and morphology of the bulk gel.

As shown in the images, the bulk gel systems retained their gelled state and mechanical consistency, indicating a uniformly continuous convex structure. There are many spherical protrusions distributed over its surface, which may contribute to the formation of DPG during milling. When the bulk gels pass through the colloid mill, the high shearing stress is applied upon the system and the shearing forces break the bulk gel into the particles with very small sizes.

**Figure 2:**
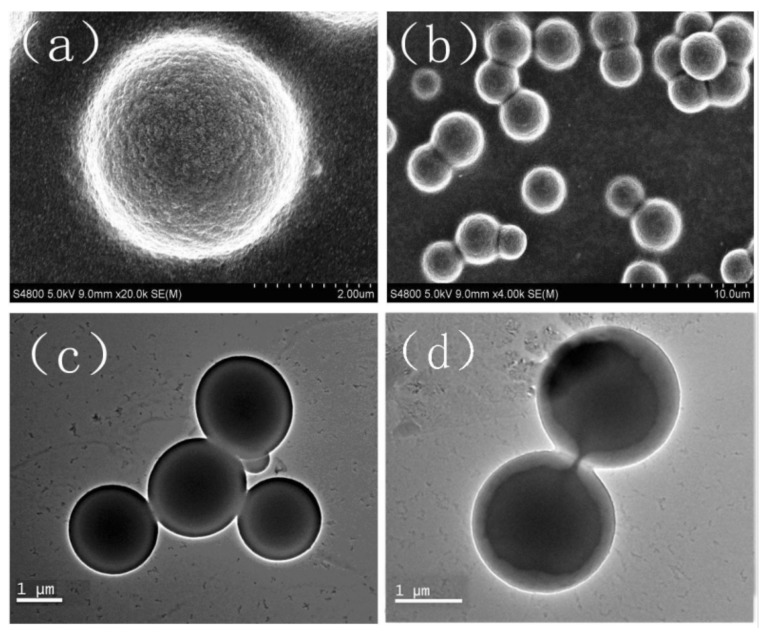
SEM (**a** and **b**) and TEM (**c** and **d**) images of the DPG particles.

Figure 2 shows the SEM and TEM images of the prepared DPG particles. It can be seen that the particles are all spherical and their sizes are uniform. The smallest size, largest size and average size of DPG particles are about 1.5 μm, 4 μm and 2.6 μm, respectively. From the image shown in Figure 2b, these particles appear to aggregate slightly, due to the high surface energy of small particles [12]. In order to better investigate the morphology of DPG particles, the TEM technique was also conducted. From the images shown in Figure 2c,d, all these DPG particles are composed of spherical particles with uniform size of about 2 μm, which is consistent with the SEM results.

**Figure 3:**
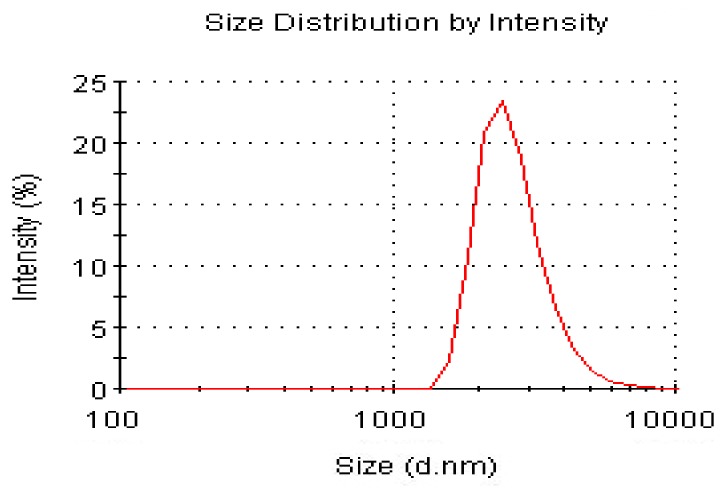
Particle size distribution of DPG.

The DLS technque is a very useful method to characterize the size distribution of DPG particles. Figure 3 shows a mass particle size distribution of DPG in the solution. The dispersion particles has a roughly 1.5–4 μm size distribution and the average size of the DPG is about 2.5 μm, which is consistent with the SEM and TEM observations.

**Figure 4:**
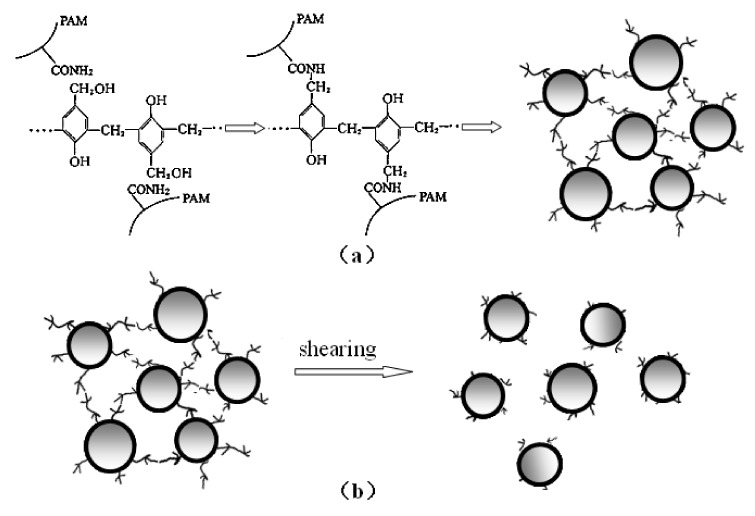
Proposed formation mechanism of the dispersed particle gel.

In order to realize a better understanding of the DPG particles formed with the high speed shearing method, a possible formation mechanism is illustrated in Figure 4. The bulk gel is formed by polymer and phenolic resin cross-linking agent at 75 °C, with many spherical protrusions distributed on the surface as shown in Figure 1. When the bulk gel is added to the colloid mill, high shearing forces, generated from the relative movement between the stator and rotor, are exerted upon the bulk gel, which makes the bulk gel change into the smaller particles. After the crushing and pulverization at the beginning, the size distribution of the produced particles varies widely, from relatively coarse to fine with the wall friction. As the shearing proceeds, the shearing forces can break the bonds between spherical protrusions and the coarse particles become uniform, resulting in the formation of DPG with regular shape and narrow particles size distribution.

## 3. Experimental

### 3.1. Materials

Nonionic polyacrylamide (PAM) with degree of hydrolysis 3.31% and average molecular weight of 9,650,000 g/mol was provided by Yuguang Co. Ltd., Dongying, China. The cross-linker of phenolic resin was purchased from Fanghua Co. Ltd., Dongying, China. The salinity of brine used in all experiments was 400 mg/L.

### 3.2. Preparation of DPG

The DPG is prepared by high speed shearing method described as follows: firstly, the gelants with 0.3% PAM and 0.6% phenolic resin cross-linking agent is put into the oven until a bulk gel is formed at 75 °C. Then water (200 g) and bulk gel (200 g) are added simultaneously to a colloid mill rotating at 3,000 rpm and milled for 3 min at 30 °C. The pale yellow solution obtained from the colloid mill were the final DPG products.

### 3.3. Measurement and Analysis

In this study, ESEM (Quanta 200 FEG, FEI Company Hillsboro, OR, USA) was used for the observation of gelation microstructure. For the preparation of samples, a drop of gel was directly placed on a covered ESEM grid, the pressure and temperature were initially set at 313~455 Pa and 0 °C with accelerating voltages of 15 kV.

Freeze-drying specimens were prepared for transmission electron microscopy (TEM) and scanning electron microscopy (SEM). Freeze-drying specimen preparation for TEM and SEM can be described as follows: a drop of the solution was placed on a copper grid and frozen at −80 °C for 2 h. Then the samples were freeze-dried in lyophilizers at −20 °C for 12 h. JEOL 100CX-II TEM and JEOL JSM-7600F SEM (JEOL Ltd., Akishima, Japan) were used to characterize the samples.

The particle size distribution is investigated by the dynamic light scattering technique (DLS, Mastersizer2000, Malvern Instruments Ltd., Worcestershire, UK).

## 4. Conclusions

The high speed shearing method for the preparation of DPG from the cross-linked gel systems within 5 minutes at room temperature has been successfully demonstrated. The DPG is composed of spherical particles with a uniform size of 2.5 μm. We expect the reported simple method will help to gain a better understanding of the preparation technology of DPG and find potential applications in the oilfield sector.
